# Infant Infections and Respiratory Symptoms in Relation to *in Utero* Arsenic Exposure in a U.S. Cohort

**DOI:** 10.1289/ehp.1409282

**Published:** 2015-09-11

**Authors:** Shohreh F. Farzan, Zhigang Li, Susan A. Korrick, Donna Spiegelman, Richard Enelow, Kari Nadeau, Emily Baker, Margaret R. Karagas

**Affiliations:** 1 Children’s Environmental Health and Disease Prevention Research Center at Dartmouth, Hanover, New Hampshire, USA; 2 Department of Epidemiology, Geisel School of Medicine at Dartmouth, Lebanon, New Hampshire, USA; 3 Channing Division of Network Medicine, Department of Medicine, Brigham and Women’s Hospital and Harvard Medical School, Boston, Massachusetts, USA; 4 Department of Environmental Health, Harvard T.H. Chan School of Public Health, Boston, Massachusetts, USA; 5 Department of Biostatistics, and; 6 Department of Epidemiology, Global Health and Nutrition, Harvard School of Public Health, Boston, Massachusetts, USA; 7 Department of Microbiology and Immunology, Geisel School of Medicine at Dartmouth, Lebanon, New Hampshire, USA; 8 Division of Immunology and Allergy, Stanford Medical School and Lucile Packard Children’s Hospital, Stanford, California, USA; 9 Department of Obstetrics and Gynecology, Geisel School of Medicine at Dartmouth, Lebanon, New Hampshire, USA

## Abstract

**Background:**

Arsenic has been linked to disrupted immune function and greater infection susceptibility in highly exposed populations. Well arsenic levels above the U.S. EPA limit occur in our U.S. study area and are of particular concern for pregnant women and infants.

**Objectives:**

We investigated whether in utero arsenic exposure affects the risk of infections and respiratory symptoms over the first year of life.

**Methods:**

We prospectively obtained information on infant infections and symptoms, including their duration and treatment (n = 412) at 4, 8, and 12 months using a parental telephone survey. Using generalized estimating equation models adjusted for potential confounders, we evaluated the association between maternal pregnancy urinary arsenic and infant infections and symptoms over the first year.

**Results:**

Each doubling of maternal urinary arsenic was related to increases in the total number of infections requiring prescription medication in the first year [relative risk (RR) = 1.1; 95% CI: 1.0, 1.2]. Urinary arsenic was related specifically to respiratory symptoms (difficulty breathing, wheezing, and cough) lasting ≥ 2 days or requiring prescription medication (RR = 1.1; 95% CI: 1.0, 1.2; and RR = 1.2; 95% CI: 1.0, 1.5, respectively), and wheezing lasting ≥ 2 days, resulting in a doctor visit or prescription medication treatment (RR = 1.3; 95% CI: 1.0, 1.7; RR = 1.3; 95% CI: 1.0, 1.8, and RR = 1.5; 95% CI: 1.0, 2.2, respectively). Associations also were observed with diarrhea (RR = 1.4; 95% CI: 1.1, 1.9) and fever resulting in a doctor visit (RR = 1.2; 95% CI: 1.0, 1.5).

**Conclusions:**

In utero arsenic exposure was associated with a higher risk of infection during the first year of life in our study population, particularly infections requiring medical treatment, and with diarrhea and respiratory symptoms.

**Citation:**

Farzan SF, Li Z, Korrick SA, Spiegelman D, Enelow R, Nadeau K, Baker E, Karagas MR. 2016. Infant infections and respiratory symptoms in relation to in utero arsenic exposure in a U.S. cohort. Environ Health Perspect 124:840–847; http://dx.doi.org/10.1289/ehp.1409282

## Introduction

Across the globe, millions are chronically exposed to drinking water containing arsenic above the 10-μg/L maximum contaminant limit set by the World Health Organization (Naujokas et al. 2013; NRC 2014; WHO 2011). Moreover, many are exposed to arsenic by diet, with common foods, such as rice, chicken, and fruit juices likely contributors to overall exposure levels (Gilbert-Diamond et al. 2011; Nachman et al. 2013; Navas-Acien and Nachman 2013). Arsenic is known for its carcinogenic potential, but growing evidence supports a role for arsenic exposure in many adverse health effects, including cardiovascular disease, diabetes, neurological effects, and immune dysfunction (ATSDR 2007; Naujokas et al. 2013; NRC 2014). At high levels of exposure, arsenic has been related to nonmalignant lung disease including bronchiectasis, chronic obstructive pulmonary disease, chronic bronchitis, and decreased lung function (Mazumder et al. 2005; Milton et al. 2001; Parvez et al. 2010, 2013; Smith et al. 2006).

Exposure to arsenic is of particular concern among pregnant women, infants, and children because they represent populations that are especially vulnerable to the health effects of environmental toxicants (Farzan et al. 2013a; Karagas 2010; Vahter 2009). Arsenic can pass from mother to fetus (Concha et al. 1998), and maternal arsenic exposure has been related to adverse pregnancy and birth outcomes, including spontaneous abortion, fetal growth restriction, and infant mortality (Hopenhayn et al. 2003; Huyck et al. 2007; Milton et al. 2005; Rahman et al. 2009, 2010; von Ehrenstein et al. 2006). Recent research suggests that maternal exposure to arsenic during pregnancy may affect an infant’s immune development and susceptibility to infections early in life (Dangleben et al. 2013). Infections are a major cause of morbidity and mortality in the first year of life (WHO 2010), including in the United States, and may have long-term impacts on children’s health. For example, infections in infancy have been related to later-life wheezing and asthma-like symptoms (Lemanske et al. 2005). In adults, arsenic exposure has been associated with impaired immune function (Smith et al. 2011), and accumulating experimental evidence indicates that it can alter immune response, viral clearance, and inflammatory responses (Kozul et al. 2009; Ramsey et al. 2013a). Consistent with this hypothesis, two prospective studies in Bangladesh reported that *in utero* arsenic exposure was related to increased rates of infant infections and alterations in immune function markers (Rahman et al. 2011; Raqib et al. 2009). Although relatively little is known about how lower levels of exposure to arsenic may affect childhood health outcomes (Farzan et al. 2013a), recent analyses from our U.S. pregnancy cohort of infants up to 4 months of age found that *in utero* arsenic exposure was associated with increased rates of respiratory infection and infections requiring prescription medication (Farzan et al. 2013b). To extend this work, we sought to investigate the extent to which *in utero* arsenic exposure may be associated with infections and other evidence of impaired immune function including early respiratory symptoms, which may indicate later-life risk of allergy and atopy (e.g., wheeze) (Ly et al. 2006; Wright 2002), among infants during their entire first year of life.

## Methods

We began recruiting 18- to 45-year-old pregnant women receiving prenatal care at study clinics in New Hampshire (USA) in January 2009, as previously described (Farzan et al. 2013b; Gilbert-Diamond et al. 2011). Briefly, women were screened for eligibility at an initial prenatal care visit and enrolled around 24–28 weeks gestation if they reported using water from a private, unregulated well in their home since their last menstrual period and were not planning a change in residence before delivery. Only singleton births were included in the cohort. All protocols were approved by the Dartmouth College Institutional Review Board. Participants provided written informed consent upon enrollment.

Participants were asked to complete a medical history and lifestyle questionnaire upon enrollment, which ascertained sociodemographic factors (age, race/ethnicity, marital status, education), reproductive history (previous pregnancies, complications, birth outcomes), and health history. Women were asked about habits, including tobacco and alcohol use, along with their home water source and consumption. At 2 weeks postpartum, mothers were sent a follow-up questionnaire to obtain additional information about pregnancy, delivery, and changes in key exposures. Participants also consented to a medical record review, which allowed additional information to be recorded about prenatal infections, medication use, birth outcomes, and delivery details and general health of the women and their infants after birth.

During the infant’s first year of life, parents were contacted to participate in three telephone surveys administered at 4, 8, and 12 months postpartum. In each survey, parents were asked a series of questions to determine whether their child had any infections (e.g., influenza, otitis media) or symptoms of infections (e.g., fever, cough, wheeze) in the preceding 4 months of life. We asked about 12 types of common infections, including colds/runny or stuffed nose, strep throat, ear infections, eye infections, whooping cough, pneumonia, bronchiolitis, respiratory syncytial virus (RSV), and influenza, as well as 5 types of symptoms, including cough, wheezing, diarrhea, and fever. For each type of infection or symptom we asked whether in the past 4 months “has [name of child] had a [infection/symptom]?” If the parent responded positively, we then asked “Did the [infection/symptom] last more than 2 days?” and “Did [name of child] see a doctor for this [infection/symptom]?” If the child had seen a doctor for the infection, we then asked “Did [name of child] receive a prescription medication for this [infection/symptom]?” The parental telephone survey responses were validated against pediatric medical records in the first year of life for a subset of the children (*n* = 153). Preliminary comparisons between the prevalence of infections involving a doctor visit obtained from pediatric medical record review were similar to those from parental interviews (data not shown).

A spot urine sample was collected from participants upon enrollment (~ 24–28 weeks gestation) and stored as previously described (Farzan et al. 2013b; Gilbert-Diamond et al. 2011). Urines were analyzed for arsenite (iAs^III^), arsenate (iAs^V^), monomethylarsonic acid (MMA), dimethylarsinic acid (DMA), and arsenobetaine by high-performance liquid chromatography (HPLC) inductively coupled plasma mass spectrometry (ICP-MS) at the University of Arizona Hazard Identification Core (Larsen et al. 1993; Le et al. 2000; Wei et al. 2001). Detection limits were 0.07–0.17 μg/L for individual species, and samples that registered below the detection limit were assigned a value equal to the detection limit divided by the square root of 2. Our primary exposure measure was total urinary arsenic during pregnancy, calculated by summing inorganic (iAs = iAs^III^ + iAs^V^) and organic (DMA, MMA) metabolites, as previously reported (Farzan et al. 2013b; Gilbert-Diamond et al. 2011). We excluded arsenobetaine from the total arsenic calculation, because it is thought to be nontoxic and to pass through the body unmetabolized (Tseng 2009). Participants also collected home water samples at the time of enrollment, which were analyzed by ICP-MS at the Dartmouth Trace Element Analysis Core, as described (Farzan et al. 2013b; Gilbert-Diamond et al. 2011).

Using natural log (ln)–transformed total urinary arsenic during pregnancy (treated as a continuous variable) as our measure of *in utero* exposure, we tested for associations with total infant infections overall, lasting ≥ 2 days, resulting in a doctor visit, or treated with prescription medication over the first year of life. We used generalized estimating equation (GEE) models (Fitzmaurice et al. 2011) for repeated measures with the log link function, compound symmetry working correlation matrix, and binomial variance, with robust variances for *p*-values (Spiegelman and Hertzmark 2005). We used the same modeling strategy to assess the relation between ln-transformed maternal urinary arsenic and specific types of common infections over the first year of life such as rhinorrhea, otitis media, RSV, upper respiratory infections (e.g., rhinorrhea, colds, nasal congestion, otitis media, conjunctivitis) or lower respiratory infections (e.g., RSV, pertussis, bronchitis, bronchiolitis, pneumonia), and acute respiratory (e.g., cough, difficulty breathing, wheeze) and gastrointestinal (e.g., diarrhea) symptoms. We further examined the interaction between arsenic exposure and each time interval (4, 8, 12 months) for the overall number of infections, as well as those lasting ≥ 2 days, resulting in a doctor visit, or treated with prescription medication. GEE models examining total infections, upper respiratory infections, lower respiratory infections, and respiratory symptoms used a Poisson distribution. All model estimates are presented in relation to ln-maternal urinary arsenic concentrations. To interpret the change in each of the outcomes per doubling of total urinary arsenic (micrograms per liter), we exponentiated the beta coefficients multiplied by the natural log of 2 [i.e., e^β × ln(2)^]. For all analyses, we used a *p*-value of 0.05 (two-sided) to define statistical significance.

In a secondary analysis, we used the same modeling strategy as above, except with an independence working correlation matrix, to assess the relation between ln-transformed maternal pregnancy urinary arsenic and common types of infections as separate outcomes within each time interval (i.e., 0–4, 5–8, and 9–12 months), similar to methods in prior work (Farzan et al. 2013b). We evaluated the relation between ln-transformed maternal urinary arsenic and total number of infections reported within each time interval, as well as those lasting ≥ 2 days, resulting in a doctor visit, or treated with a prescription medication.

Models included covariates that could influence infection risk based on *a priori* considerations, including age at enrollment (years), smoking during pregnancy (yes/no), relationship status (married, single, divorced/widowed), education (≤ 11th grade, high school, some college, college, postgraduate), parity (0, 1–2, ≥ 3), delivery type (vaginal, cesarean), infant sex, birth weight (grams), gestational age (weeks), breastfeeding (ever, never in GEE models; yes/no during each interval for time specific models), and child care attendance (yes/no during each interval). Gestational age was calculated using first trimester ultrasound gestational age estimates or, if an ultrasound was unavailable, last menstrual period date. For maternal smoking and birth weight, which were missing for 29 and 15 individuals, respectively, due to incomplete records at the time of analysis, we applied the missing indicator method in our GEE analyses (Miettinen 1985).

Finally, we assessed nonlinear trends of the data for the total number of infections in the first year resulting in a doctor visit or treated with a prescription medication using a generalized additive model (GAM) with cubic regression splines.

## Results

Of the 1,033 mothers enrolled in our study, 726 had infants who were at least 12 months of age at the time of analysis. Three hundred seven women either had not yet given birth or had yet to participate in an interval questionnaire. A total of 683 mothers had completed at least one follow-up questionnaire during the infant’s first 12 months, and 412 had maternal urinary arsenic measured at the time of these analyses. Mothers who had urinary arsenic results available did not significantly differ from those who did not (*n* = 271) in any key demographic or lifestyle characteristics, nor did mothers who completed at least one questionnaire differ from those who had not (*n* = 350) (data not shown).

***Demographic data.*** The mean (± SD) participant age was 31.2 ± 4.9 years at the time of delivery (Table 1). Most (95%) reported that they did not smoke while pregnant and were not exposed to secondhand smoke (91%). Slightly more than half of infants were female (54%) and infants had a mean (± SD) birth weight of 3,438 ± 544 g. The average (± SD) gestational age at birth was 40 ± 2 weeks (Table 1). Most children (70%) were not in child care at 4 months, but the percentage of children receiving all care in the home decreased with age (63% at 8 months, 60% at 12 months). Forty-three percent of mothers reported exclusive breastfeeding at 4 months, and 36% were still were breastfeeding their child at 12 months (Table 1).

**Table 1:** Selected sample characteristics for mothers and infants participating in the New Hampshire Birth Cohort Study (*n *= 412).

Variable	Mean (range) or percent
Maternal variables
Drinking-water arsenic^*a*^	4.6 (0.0–147.7)
Median (IQR)	0.5 (3.1)
Pregnancy urinary arsenic	5.7 (0.5–58.3)
Median (IQR)	3.8 (4.8)
Age at enrollment (years)	31.2 (18.4–44.5)
< 20	2
20–29	40
30–35	41
> 35	17
Education level^*a*^
< 11th grade	1
High school graduate, GED	9
Junior college, some college, technical school	21
College graduate	39
Postgraduate schooling	30
Relationship status^*a*^
Single	12
Married	84
Separated, divorced	4
Smoking during pregnancy^*a*^
Yes	5
Secondhand smoke exposure^*a*^
Yes	9
Prepregnancy BMI^*a*^	25.1 (17.0–48.3)
Parity^*a*^
Nulliparous	43
1–2	50
≥ 3	7
Delivery type^*a*^	
Vaginal (spontaneous or induced)	67
Cesarean	33
Infant variables
Sex^*a*^
Male	46
Birth weight^*a*^ (g)	3438.3 (1380.0–5318.0)
Gestational age^*a*^ (weeks)	39.5 (26.9–44.9)
Child care setting
In child care at 4 months	30
In child care at 8 months	37
In child care at 12 months	40
Feeding
Exclusively formula fed at 4 months	7
Exclusively breastfed at 4 months	43
Still breastfeeding at 8 months	56
Still breastfeeding at 12 months	36
Infections within first 12 months of life
At least one infection	94
At least one infection lasting ≥ 2 days	90
At least one infection resulting in a doctor visit	51
At least one infection treated with prescription medication	41
IQR, interquartile range. ^***a***^Sum of subjects is less than total sample size due to missing values (25 subjects missing drinking-water arsenic, 33 missing education and relationship status, 29 missing smoking, 35 missing prepregnancy BMI and secondhand smoke exposure, 1 missing parity, 11 missing delivery type, 2 missing sex, 15 missing birth weight, and 10 missing gestational age).	

Infections were common, with nearly all parents (94%) reporting at least one infection in the infant’s first year of life, of which 90% lasted ≥ 2 days (Table 2). More than half (51%) reported at least one infection that involved a doctor visit, and 41% reported at least one infection that was treated with prescription medicine. Upper respiratory infections were the most commonly reported type of infection (89% of infants during the first year).

**Table 2:** Relative risk estimates*^a^* (95% CI) for infant infections or symptoms lasting ≥ 2 days in the first year of life, per doubling of maternal ~ 24–28 weeks gestation urinary arsenic (*n *= 412).

Infections	0–4 months	5–8 months	9–12 months	Over the first year
Any infection lasting ≥ 2 days	1.1 (1.0, 1.2)	1.0 (0.9, 1.1)	1.0 (0.9, 1.1)	1.0 (0.9, 1.1)
*n*^*b*^	207	229	246	349
Respiratory tract infections (RTI)
Any upper RTI	1.1 (1.0, 1.2)	1.0 (0.9, 1.1)	1.0 (0.9, 1.1)	1.0 (1.0, 1.1)
*n*^*b*^	206	228	246	349
Any lower RTI (i.e., bronchitis, pneumonia, bronchiolitis, RSV, pertussis)	1.2 (0.8, 1.8)	1.3 (0.9, 1.8)	1.0 (0.6, 1.8)	1.1 (0.9, 1.4)
*n*^*b*^	15	31	10	53
Acute symptoms, conditions, illnesses
Any respiratory (i.e., cough, wheeze, difficulty breathing)	1.2 (1.0, 1.4)	1.0 (0.9, 1.2)	1.2 (1.0, 1.5)	1.1 (1.0, 1.5)
*n*^*b*^	103	126	104	231
Wheezing	1.4 (0.9, 2.3)	1.6 (1.0, 2.4)	0.7 (0.4, 1.4)	1.3 (1.0, 1.7)
*n*^*b*^	19	22	10	47
Cough	1.2 (1.0, 1.5)	1.0 (0.8, 1.2)	1.2 (1.0, 1.5)	1.1 (1.0, 1.2)
*n*^*b*^	92	107	98	220
Difficulty breathing	1.0 (0.6, 1.8)	1.2 (0.8, 1.9)	1.3 (0.7, 2.7)	1.1 (0.8, 1.5)
*n*^*b*^	14	20	8	41
Gastrointestinal (i.e., diarrhea)	1.6 (0.9, 2.9)	1.3 (0.9, 2.0)	1.1 (0.8, 1.5)	1.2 (0.9, 1.6)
*n*^*b*^	13	28	43	70
Fever	1.2 (0.6, 2.2)	1.1 (0.8, 1.5)	1.2 (0.9, 1.6)	1.1 (0.9, 1.3)
*n*^*b*^	12	35	54	92
^***a***^Estimates after adjustment for maternal age, parity, smoking, infant sex, gestational age, birth weight, breastfeeding, and child care attendance. ^***b***^Number of children with a report of at least one infection (for estimates over the first year, each child could contribute up to three reports, one per interval questionnaire, of any type of infection).				

***Arsenic exposure.*** The median maternal total urinary arsenic concentration was 3.8 μg/L and the mean (± SD) was 5.7 ± 6.5 μg/L (range, 0.5–58.3 μg/L) (Table 1). The average drinking water arsenic concentration was 4.6 μg/L (range, 0.0–147.7 μg/L) (Table 1).

**Figure 1:**
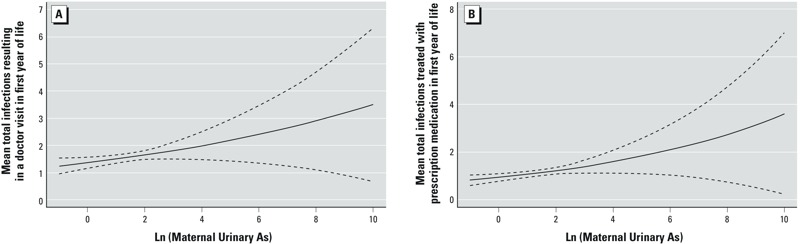
Maternal arsenic exposure and mean total infections over the first year of life. The relation of ln-transformed maternal urinary arsenic at ~ 24–28 weeks gestation with mean total infections over the first year of life that resulted in a doctor visit (*A*) or treatment with prescription medication (*B*), based on Poisson models, adjusted for maternal age, parity, smoking, infant sex, gestational age, birth weight, breastfeeding, and child care attendance. *p*-Values for linearity based on GAM were 0.39 and 0.73, respectively. Dotted lines represent the 95% CI.

**In utero *arsenic exposure and infant infections.*** After adjustment for maternal age, parity, smoking, infant sex, gestational age, birth weight, breastfeeding, and child care attendance, each doubling of maternal urinary arsenic concentration during pregnancy on a micrograms per liter natural log scale was associated with an increased risk of any infection resulting in a doctor visit [relative risk (RR) = 1.1; 95% confidence interval (CI): 1.0, 1.2] (Table 3, Figure 1A) or that was treated with prescription medication (RR = 1.1; 95% CI: 1.0, 1.2) (Table 4, Figure 1B). We did not find evidence of nonlinearity (e.g., *p*-value for linearity = 0.73 for infections treated with prescription medication). Each doubling of maternal urinary arsenic was associated with increased risk of infections treated with prescription medication (Table 4), including upper respiratory (RR = 1.1; 95% CI: 1.0, 1.2), lower respiratory (RR = 1.2; 95% CI: 1.0, 1.5), and colds, or runny or stuffed noses (RR = 1.2; 95% CI: 1.0, 1.4). Maternal urinary arsenic was associated with greater risk of respiratory symptoms treated with prescription medication (RR = 1.2; 95% CI: 1.0, 1.5) (Table 4), as well as for those lasting ≥ 2 days (RR = 1.1; 95% CI: 1.0, 1.5) (Table 2). Maternal urinary arsenic was associated with an increased risk of wheezing lasting ≥ 2 days (RR = 1.3; 95% CI: 1.0, 1.7), resulting in a doctor visit (RR: 1.3, 95% CI: 1.0, 1.8) or treatment with prescription medication (RR = 1.5; 95% CI: 1.0, 2.2) (Tables 2, 3, and 4). Additionally, diarrhea resulting in a doctor visit (RR = 1.4; 95% CI: 1.1, 1.9) in the first year was associated with arsenic exposure, as was fever resulting in a doctor visit (RR = 1.2; 95% CI: 1.0, 1.5) (Table 3).

**Table 3:** Relative risk estimates*^a^* (95% CI) for infant infections or symptoms resulting in a doctor visit in the first year of life, per doubling of maternal ~ 24–28 weeks gestation urinary arsenic (*n *= 412).

Infections	0–4 months	5–8 months	9–12 months	Over the first year
Any infection resulting in a doctor visit	1.1 (1.0, 1.2)	1.1 (1.0, 1.2)	1.0 (0.9, 1.2)	1.1 (1.0, 1.2)
*n*^*b*^	97	125	135	197
Respiratory tract infections (RTI)
Any upper RTI	1.1 (1.0, 1.3)	1.1 (0.9, 1.2)	1.0 (0.9, 1.2)	1.1 (1.0, 1.1)
*n*^*b*^	96	123	133	197
Any lower RTI (i.e., bronchitis, pneumonia, bronchiolitis, RSV, pertussis)	1.0 (0.7, 1.4)	1.2 (0.9, 1.6)	1.0 (0.6, 1.6)	1.1 (0.9, 1.4)
*n*^*b*^	18	33	11	49
Acute symptoms, conditions, illnesses
Any respiratory (i.e., cough, wheeze, difficulty breathing)	1.2 (1.0, 1.4)	1.0 (0.9, 1.2)	1.1 (0.9, 1.4)	1.1 (0.8, 1.3)
*n*^*b*^	54	63	38	107
Wheezing	1.5 (0.9, 2.5)	1.5 (0.9, 2.4)	1.0 (0.6, 1.7)	1.3 (1.0, 1.8)
*n*^*b*^	15	19	10	46
Cough	1.2 (0.9, 1.6)	0.9 (0.7, 1.2)	1.2 (0.9, 1.7)	1.0 (0.9, 1.2)
*n*^*b*^	47	47	33	126
Difficulty breathing	1.5 (0.9, 2.6)	1.2 (0.8, 1.8)	1.5 (0.8, 2.9)	1.3 (0.9, 1.8)
*n*^*b*^	15	20	10	48
Gastrointestinal (i.e., diarrhea)	1.9 (1.1, 4.8)	1.5 (0.9, 2.5)	1.3 (0.9, 1.9)	1.4 (1.1, 1.9)
*n*^*b*^	7	10	17	34
Fever	1.2 (0.7, 1.9)	1.4 (1.0, 1.9)	1.2 (0.9, 1.6)	1.2 (1.0, 1.5)
*n*^*b*^	19	36	51	108
^***a***^Estimates after adjustment for maternal age, parity, smoking, infant sex, gestational age, birth weight, breastfeeding, and child care attendance. ^***b***^Number of children with a report of at least one infection (for estimates over the first year, each child could contribute up to three reports, one per interval questionnaire, of any type of infection).				

**Table 4:** Relative risk estimates*^a^* (95% CI) for infant infections or symptoms treated with prescription medication**in the first year of life, per doubling of maternal (~ 24–28 weeks gestation) urinary arsenic (*n *= 412).

Infections	0–4 months	5–8 months	9–12 months	Over the first year
Any infection treated with prescription medication	1.3 (1.1, 1.5)	1.0 (0.9, 1.2)	1.1 (0.9, 1.2)	1.1 (1.0, 1.2)
*n*^*b*^	55	90	108	157
Respiratory tract infections (RTI)
Any upper RTI	1.2 (1.0, 1.5)	1.0 (0.9, 1.2)	1.1 (0.9, 1.2)	1.1 (1.0, 1.2)
*n*^*b*^	53	84	106	154
Any lower RTI (i.e., bronchitis, pneumonia, bronchiolitis, RSV, pertussis)	1.6 (1.1, 2.3)	1.1 (0.8, 1.5)	1.1 (0.7, 1.9)	1.2 (1.0, 1.5)
*n*^*b*^	14	26	10	40
Acute symptoms, conditions, illnesses
Any respiratory (i.e., cough, wheeze, difficulty breathing)	1.5 (1.1, 2.0)	1.1 (0.9, 1.3)	1.3 (1.0, 1.6)	1.2 (1.0, 1.5)
*n*^*b*^	16	35	23	55
Wheezing	2.1 (1.0, 4.3)	1.4 (0.8, 2.5)	1.0 (0.5, 2.0)	1.5 (1.0, 2.2)
*n*^*b*^	9	17	7	32
Cough	1.5 (0.8, 2.9)	0.9 (0.6, 1.4)	1.4 (0.8, 2.2)	1.2 (0.9, 1.6)
*n*^*b*^	13	26	21	53
Difficulty breathing	1.6 (0.9, 1.3)	1.2 (0.8, 1.8)	1.5 (0.8, 2.9)	1.2 (0.9, 1.6)
*n*^*b*^	7	17	8	29
Gastrointestinal (i.e., diarrhea)	—	—	—	—
*n*^*b*^	1	1	3	3
Fever	1.3 (0.7, 2.2)	1.1 (0.7, 1.7)	1.3 (0.8, 1.9)	1.2 (0.9, 1.6)
*n*^*b*^	8	25	33	61
—, too few observations to estimate. ^***a***^Estimates after adjustment for maternal age, parity, smoking, sex, gestational age, birth weight, breastfeeding, and child care. ^***b***^Number of children with at least one reported infection (Over the first year, each child could contribute up to three reports, one per interval, for any infection).				

In general, associations with arsenic exposure during pregnancy were stronger for infections at 4 months of age and weaker for infections or symptoms at 8 or 12 months. Over the first 4 months, maternal urinary arsenic was associated with an increased risk of total infections resulting in a doctor visit (RR = 1.1; 95% CI: 1.0, 1.2) or treatment with prescription medication (RR = 1.3; 95% CI: 1.1, 1.5), upper respiratory infections resulting in a doctor visit (RR = 1.1; 95% CI: 1.0, 1.3) or treatment with prescription medication (RR = 1.2; 95% CI: 1.0, 1.5), and lower respiratory infections treated with prescription medication (RR = 1.6; 95% CI: 1.1, 2.3) (Tables 3 and 4). Urinary arsenic related to specific symptoms in the first 4 months, including diarrhea resulting in a doctor visit (RR = 1.9; 95% CI: 1.1, 4.8), wheezing treated with prescription medication (RR = 2.1; 95% CI: 1.0, 4.3), and any respiratory symptom resulting in a doctor visit (RR = 1.2; 95% CI: 1.0, 1.4) or prescription medication (RR = 1.5; 95% CI: 1.1, 2.0) (Tables 3 and 4). At 8 months, maternal urinary arsenic was associated with an increased risk of total infections resulting in a doctor visit (RR = 1.1; 95% CI: 1.0, 1.2) and wheezing lasting ≥ 2 days (RR = 1.6; 95% CI: 1.0, 2.4) (Tables 2 and 3). At 12 months of age, maternal urinary arsenic was related to an increased risk of respiratory symptoms treated with prescription medication (RR = 1.3; 95% CI: 1.0, 1.6) (Table 4), and cough lasting ≥ 2 days (RR = 1.2; 95% CI: 1.0, 1.5) (Table 2).

## Discussion

Prenatal arsenic exposure was associated with an increased risk of infections among children in the first year of life in our U.S. cohort, particularly respiratory infections and symptoms that require a doctor visit or treatment with prescription medication. Associations were generally strongest within the first 4 months, when *in utero* arsenic exposure was the most consistently associated with upper and lower respiratory infections as well as diarrhea, suggesting that the early postnatal period may be an especially vulnerable period for arsenic’s effects (Dietert and Piepenbrink 2006). Associations were most consistent for reported infections and symptoms that required a doctor visit or prescription medication, which could reflect either more accurate reporting of these outcomes or stronger associations with more severe disease or symptoms.

Although studies of early-life arsenic exposure in relation to childhood infections in U.S. populations are lacking, our findings parallel those observed among more highly exposed children elsewhere in the world (Rahman et al. 2011; Raqib et al. 2009). In Bangladesh, maternal arsenic exposure during pregnancy was related to an increased risk of acute respiratory infection in the first year of life in male infants, as well as increased risks of maternal fever and diarrhea during pregnancy, suggesting potential arsenic-related immune effects for both mother and child (Raqib et al. 2009). A larger cohort study (*n* = 1,552) in Bangladesh likewise found that higher levels of urinary arsenic in pregnancy were associated with increased risk of lower respiratory infections and diarrhea in infants over the first year of life (Rahman et al. 2011). In our earlier analysis of *in utero* arsenic exposure and infections and symptoms up to 4 months of age in a smaller subset of infants, we found that maternal urinary arsenic was related to total number of infections requiring a doctor visit or prescription medication, as well as respiratory infections and symptoms treated with prescription medication in the first 4 months (Farzan et al. 2013b). In the present study, in which we expanded our sample size and obtained multiple repeated measurements of infections through age 1 year, we found an association between maternal arsenic exposure during pregnancy and increased risks of total infections, fever, and diarrhea resulting in a doctor visit, as well as infections and symptoms treated with prescription medication, including respiratory infections, respiratory symptoms, and wheezing. Findings from the present study are consistent with the results of previous studies, which have consistently observed increases in similar types of infections in the first year of life, most frequently respiratory infections, across a range of exposure levels.

Although it is possible that associations between *in utero* arsenic exposure and early infections in our study population represent a transient effect, prenatal arsenic exposure has been associated with immune alterations that may indicate long-term impacts on immune functionality. These include decreased thymic size and function, enhanced inflammatory responses, increased oxidative stress and cytokine levels, and immune changes in the placenta (Ahmed et al. 2011, 2012; Dangleben et al. 2013; Fry et al. 2007; Rager et al. 2014; Raqib et al. 2009). Evidence suggests that arsenic exposure may fundamentally transform the immune response by altering developmental signaling pathways. Among newborns in the BEAR (Biomarkers of Exposure to Arsenic) cohort in Mexico, prenatal arsenic exposure was associated with altered cord blood expression levels of 12 microRNAs and 334 mRNA transcripts (Rager et al. 2014). Pathway analysis and interaction mapping found that many of these molecules are involved in innate and adaptive immune response, as well as respiratory disease (Rager et al. 2014), similar to previously observed inflammatory and immune-related gene alterations in arsenic-exposed newborns in Thailand (Fry et al. 2007). Recent evidence indicates that even relatively low levels of *in utero* arsenic exposure can impair T-cell function and alter the fetal immune cell repertoire found in cord blood at birth, skewing it toward a pro-inflammatory Th2 (T-helper 2) phenotype (Nadeau et al. 2014), which could impact long-term immune response and allergy development (Belderbos et al. 2009). Although limited in number, these studies begin to indicate that prenatal arsenic exposure may impair healthy immune development, although further mechanistic data are needed, especially at lower exposure levels.

A growing body of evidence supports a connection between arsenic exposure and lung disease and impairment. In animal models, transplacental arsenic exposure affects lung development, by altering pulmonary structure and function (Lantz et al. 2009; Ramsey et al. 2013b), changing expression of lung morphogenesis and structurally important extracellular matrix genes (Petrick et al. 2009; Ramsey et al. 2013b), and increasing susceptibility to infection (Ramsey et al. 2013a). Studies from Bangladesh and West Bengal described increased incidence of respiratory disorders, chronic bronchitis, decreased lung function, and bronchiectasis among arsenic-exposed individuals, compared with unexposed adults (Mazumder et al. 2000, 2005; Milton et al. 2001; Milton and Rahman 2002; von Ehrenstein et al. 2005). In Antofogasta, Chile, where public water arsenic levels reached nearly 900 μg/L from 1958 to 1971, residents experienced increased rates of mortality from pulmonary tuberculosis (Smith et al. 2011), and those exposed to high arsenic levels *in utero* or during early life had higher mortality from lung cancer, bronchiectasis, and chronic lung disease, than residents of a nonexposed region (Smith et al. 2006). Prospective work from Bangladesh found that well and urinary arsenic were related to increases in respiratory symptoms, including chronic cough and difficulty breathing, as well as significant lung function impairment (Parvez et al. 2010, 2013). Similar associations between high-level early life arsenic exposure and respiratory impairment in children have also been reported. In Bangladesh, 7- to 17-year-olds exposed to > 500 μg/L water arsenic throughout childhood, and likely *in utero* as well, reported increased wheezing [odds ratio (OR) = 8.4; 95% CI: 1.7, 42.6] and shortness of breath (OR = 3.9; 95% CI: 1.1, 13.7), compared with children exposed to water with arsenic < 10 μg/L (Smith et al. 2013). A recent prospective study of 6- to 12-year-old Mexican children reported a relationship between *in utero* and early-life arsenic exposure and clinical indicators of decreased lung function (Recio-Vega et al. 2015). These studies indicate that arsenic exposure across life stages may adversely affect lung function and increase risk of lung disease. Although additional follow-up is needed, our findings of increased risk of wheezing or respiratory symptoms may signal later risk of lung disease (Ly et al. 2006; Wright 2002).

Our study has strengths and limitations. Our analyses used carefully collected prospective data, including repeated assessments of infection occurrence over the first year of life, as well as information on potential confounders, including detailed maternal medical and sociodemographic information. The study’s internal validity is strengthened by the prospective longitudinal design. Repeated-measures analyses can reveal changes in the frequency of common outcomes that may appear small on an individual basis, but are relevant to the population at large (Farrington 1991). Our exposure measure was maternal urinary arsenic, a biomarker of *in utero* exposure. However, we lacked sufficient information on postnatal infant exposure to arsenic (i.e., from food or water sources), which may contribute to overall exposure and health outcomes as these children age. The accuracy of parental recall is a potential source of bias, but we attempted to minimize misclassification and assess infection severity by focusing on infections requiring a doctor visit or prescription medicine. We cannot rule out the possibility that reporting inaccuracies may be related to maternal exposure status, potentially causing differential misclassification, or of nondifferential misclassification reducing our ability to observe associations.

## Conclusions

Infectious diseases still remain a primary cause of mortality in young children, resulting in > 4 million deaths before the age of 5 each year (WHO 2010). All infants, even those born in developed countries, experience a high burden of infection-related morbidity and mortality, particularly in the first year of life and primarily from respiratory infections and diarrhea (Mehal et al. 2012; Tregoning and Schwarze 2010; Yorita et al. 2008). Early-life respiratory infections have been associated with wheezing symptoms, and may predict later-life asthma and atopic disease (Ly et al. 2006; Wright 2002). Incidence of these conditions has risen rapidly in recent years (Aberg et al. 1995; Heinrich et al. 2002), with approximately 300 million individuals worldwide affected by asthma and approximately 30% of the population of industrialized countries affected by atopic conditions (Palomares et al. 2010; WHO 2007). Moreover, common rhinovirus infection was the strongest predictor of later-life wheezing among children at high risk of developing asthma (Lemanske et al. 2005). Although our current knowledge of the effects of arsenic exposure on childhood immunity is still very limited, our study is among the first to explore this issue in a population exposed at relatively common environmental levels. Millions worldwide are exposed to elevated arsenic concentrations in drinking water, and dietary sources may contribute to overall exposure; thus even small increases in infection morbidity or severity due to arsenic exposure could have broad public health impacts.
